# Risk Analysis by a Probabilistic Model of the Measurement Process

**DOI:** 10.3390/s21062053

**Published:** 2021-03-15

**Authors:** Wojciech Toczek, Janusz Smulko

**Affiliations:** Faculty of Electronics, Telecommunication and Informatics, Gdańsk University of Technology, 80-233 Gdańsk, Poland; jsmulko@eti.pg.edu.pl

**Keywords:** mathematical model, sensor sub-process, reconstruction sub-process, measurement uncertainty, risk analysis

## Abstract

The aim of the article is presentation of the testing methodology and results of examination the probabilistic model of the measurement process. The case study concerns the determination of the risk of an incorrect decision in the assessment of the compliance of products by measurement. Measurand is characterized by the generalized Rayleigh distribution. The model of the measurement process was tested in parallel mode by six risk metrics. An undesirable effect in the reconstruction building block of the model was detected, consisting in the distortion of probability distribution at the edges of the measuring range. The paper gives guidelines on how to use the model, to obtain the analytical risk assessment consistent with the results of the Monte Carlo method. The study can be useful in product quality control, test design, and fault diagnosis.

## 1. Introduction

The Joint Committee for Guides in Metrology issued the document JCGM 106:2012 [1] dedicated to the role of measurement uncertainty in deciding conformance to the relevant standards or specified technical requirements of products, processes, systems, and materials. Conformity assessment is an area of importance in manufacturing quality control, legal metrology, and in the maintenance of health and safety [2]. The audience of this document includes quality managers, members of standards development organizations, accreditation authorities and the staffs of testing and measuring laboratories, inspection bodies, certification bodies, regulatory agencies, and researchers [1]. Section 9 of [1] is particularly useful for analyzing the risk of an erroneous decision based on a measurement result close to the tolerance limit, which cannot be repeated due to the passage of time or the disappearance of the measured phenomenon.

Methods of risk evaluation are based on the joint probability density function characterizing a measurable property of the item of interest, and the measurement process. Methods of assigning a prior probability density function for a measurand are discussed in Annex B to [1]. About the measurement process, it is said “The form of a model is assigned based on the design of the measuring system, information supplied by calibrations and knowledge of relevant influence quantities”. Readers are forced to look for such probabilistic models in the literature of measurement science.

In many papers [3,4,5,6,7,8,9,10,11,12,13,14,15,16,17,18,19,20,21] authors regard any measurement process as a concatenation of two sub-processes, namely, sensor process and reconstruction process. Sensor process is the physical process of producing an observable output by transferring measurement information into easily interpretable phenomena, typically electrical, or optical ones. Reconstruction constitutes the determination of the measurand, given as numbers, from the observable output. The simplest reconstruction process is reading of the scale of an indicating instrument by the operator [4,14]. In this case, the measurand is reconstructed based on the visual comparison of the image of the pointer with the image of the scale. It is worth noting that the sub-processes names “conversion” and “reconstruction” [4] or “observation” and “restitution” [5] are also used, respectively.

In the probabilistic approach to the analysis of measurement processes [5,6,12] the output of the measuring system is treated as a random variable *X_m_*, whose value is not clearly determined by the measurand *X* but is characterized by the conditional probability distribution.

In work [22] authors implemented a probabilistic measurement model in the Matlab environment and used it to optimize a built-in self-tester for a fully differential electronic circuit. The model was used for setting test limits under production process deviations as a trade-off between defect level and yield loss. During this previous work, some discrepancies were found between the results of the analytical calculations and the results of the Monte Carlo simulation. It prompted the authors to thoroughly examine the properties of the model, find the sources of errors and remove them. The methodology of testing the model was applied consisting in calculation the set of risk measures increased to six, not only two as previously. The research was run based on a case study, additionally using, as a reference, the Monte Carlo method with a large number of trials. As a result of the tests, an undesirable effect in the reconstruction sub-process was detected, consisting in the distortion of probability distributions at the ends of the measuring range. This effect is amplified with increasing measurement uncertainty, causing errors in risk assessment.

The plan of the paper is as follows. The definitions of the metrics of the risk of an incorrect decision, due to measurement uncertainty, were systematized in Section 2. Section 3 presents in short the probabilistic model of the measurement process and its numerical reference standard. In Section 4, verification of the model was carried out using case study. An exemplary production testing process was studied, in which the voltage magnitude was a measured parameter. Section 5 explains the mechanism of formation of distortions of probabilistic distributions and proposes a way to eliminate them. Numerical results of testing the model for the case study are also presented. Finally, discussion and some general conclusions are given in Section 6 and Section 7.

## 2. Risk of Incorrect Decision Due to Measurement Uncertainty

The content of this section is known [23,24,25,26] and it does not convey any original novelty but is necessary for a full description of the methodology used for testing the probabilistic model of the measurement process.

The measurement uncertainty is a parameter that characterizes the dispersion of values that can be reasonably assigned to the measured quantity. The measurement uncertainty considered in this paper is interpreted as a standard deviation of the random variable describing the state of knowledge about a measurement process.

Conformity to a specified requirement means that a feature of an item of interest lies within the tolerance interval *L*. The item, whose property *x* conforms to the specified requirement (x∈L) is good (*G*), otherwise (x∉L) it is bad (*B*). The status of an item is checked during testing, which is the combination of the measurement and decision processes.

Usually, a binary decision rule that concerns acceptance or rejection of the unit under test is used. There are four possible outcomes of a conformity assessment inspection (Table 1). If the measurement process was perfectly accurate, then it would accept (*A*) the good items $\left(x_{m}\in D\right)$ and reject (*R*) the bad items $\left(x_{m}\notin D\right)$, based on measured quantity value $x_{m}$ and acceptance interval *D*. The influence of measurement uncertainty leads to the risk of mistake in deciding whether an item conforms to a specified requirement. Such mistakes are of two types: the item is rejected but conforms with the specification, or the item is accepted but does not conform with the specification.

The risk of false rejection has traditionally been called the producer’s risk, while the risk of false acceptance has been called the consumer’s risk. By conditioning the probability on the status of the unit under test (good or bad), one obtains four conditional probabilities characterizing the test (Table 2).

By conditioning the probability on the test result (accepted or rejected), one obtains four conditional probabilities, characterizing the quality of an item after testing (Table 3).

Analysis of Table 1, Table 2 and Table 3 leads to the conclusion that the producer’s risk metrics may be two kinds of probabilities: joint *P*(*R*,*G*), and conditional *P*(*R*|*G*), *P*(*G*|*R*), and the consumer’s risk metrics the probabilities: joint *P*(*A*,*B*), and conditional *P*(*B*|*A*), *P*(*A*|*B*).

The risk metrics, specified in Table 1, Table 2 and Table 3, are used in the subsequent sections to test the probabilistic model of the measurement process, to assess its performance.

## 3. Probabilistic Models of the Measurement Process

A measurement is a process of experimentally determining one or more values that can reasonably be attributed to the selected quantity [27]. When we perform a measurement, we get an observation *Y*, based on which, we can identify the value of the measurand *X*. The description of the sensor sub-process is provided by the conditional probability distribution

(1) p(y|x)

that is, the probability distribution of *Y*, given the value *x* of the measurand *X*. The accuracy of the probability distribution (1) depends on the sophistication of the sensor design, protection against environmental factors, and on the removal of method errors.

Reconstruction sub-process may be described as the probabilistic inversion of the transformation defining the sensor sub-process [3,5,6,8,10,12]. Such an inversion is performed with Bayes’ theorem, using a non-informative prior, that is, a prior with minimal influence on the inference. Rossi in [5] recommends using a flat (uniform) prior, according to the Bayes-Laplace postulate. Although a priori information on measurand *X* is available, at this stage of calculation, a uniform distribution is assumed to obtain a posterior distribution only based on the probabilistic characteristics of the sensor process. For the uniform prior distribution, we can approximate the results from Bayes’ theorem by substituting a constant for the prior distribution so that

(2) $$p\left(x|y\right)=\frac{p\left(y|x\right)}{\int_{L_{X}}p\left(y|x\right)dx}$$

where $L_{x}$ is the admissible range of values for *X*.

The best estimate of *X*, constituting a single measurement value, is the expectation $x_{m}=E\left(X|y\right)$ of distribution (2). The measurement value is also a random variable $X_{m}$, since it is not uniquely determined by the measurand *X* but is characterized by the conditional probability distribution $p\left(x_{m}|x\right)$, obtained by concatenation of the results of two modeling steps. The reconstruction of a measurand and concatenation of two subprocesses is simultaneously performed by integration of distribution *p*(*y*|*x*), with respect to *Y*, using a random variable transformation theorem [28]

(3) $$p\left(x_{m}|x\right)=\int_{D_{Y}}\delta \left[x_{m}-E\left(X|y\right)\right]\;p\left(y|x\right)dy$$

where $D_{Y}$—the set of possible values for *Y*, and δ—the Dirac delta operator.

Distribution (3) describes the overall measurement process, relating the measurement value $X_{m}$ to the measurand *X* using general definition of $x_{m}$. In many cases it may be possible to express $x_{m}$ directly in terms of *y* [12].

### Numerical Reference Standard

The Monte Carlo (MC) method was used as the numerical reference standard for model verification. Many approaches have been explored in the literature toward estimation of performance metrics, with ppm precision, for electronic circuits [29,30,31,32,33,34]. Statistical model of the electronic circuit that can be simulated very fast is crucial to achieve them in reasonable time. In [29], the proposed method relies on estimating the joint probability density function (pdf), which is subsequently sampled to rapidly generate a large volume of new data. In [30] authors used a very fast statistical simulation called statistical blockade [31]. The key idea of the method [32] is to build a statistical model of the tested circuit using nonparametric adaptive kernel density estimation. In our paper the statistical model of the circuit under test, used in case study, was derived analytically.

Using the statistical model, risk assessment consists in counting the number of results of a computer experiment and dividing the sum by the size of the population of results or a subset of this population. For example, the risk *P*(*B*|*A*) is determined by counting the results of simulations belonging to the region of incorrect acceptance and dividing it by the sum of the results falling within acceptance range *D*. When determining the risk *P*(*R*|*G*), we count the results belonging to the region of incorrect rejection and divide it by the sum of results within the tolerance interval *L*.

Contemporary personal computers allow the calculation of risk by the MC method with a large number of trials, in reasonable time. This paper presents the probabilities evaluated after 10^9^ trials.

## 4. Case Study

For a given production process and measurement system used, knowledge about the possible results of checking the compliance of a product, selected at random from the population, is contained in the joint probability distribution of the measurement result and the measurand [1]. Deriving this distribution requires knowledge of the probability distributions that characterize the measurement process and the measurand.

Verification of the model of measurement process was carried out for an example of production testing using voltage magnitude as the measured parameter. Suppose that it is a critical parameter of production process. Such a measurand is characterized by the Rayleigh distribution, which became widely known in communication theory for describing instantaneous peak power of radio signals. It has received a considerable attention from engineers and physicists for modelling wave propagation, radiation, synthetic aperture radar images, and other related phenomena [35].

Let *x* denote the measured voltage magnitude (measurand), $x_{m}$ denote the result of measurement, and $L_{u}$ the specified upper tolerance limit of the parameter *x*. The generalized Rayleigh distribution *p*(*x*) is assigned to characterize the measurand

(4) $$p\left(x\right)=\frac{x}{\sigma_{\alpha }\sigma_{\beta }\sqrt{1-r^{2}}}e^{-\frac{x^{2}}{2\left(1-r^{2}\right)}a}I_{0}\left(\frac{x^{2}}{2\left(1-r^{2}\right)}\sqrt{b^{2}+c^{2}}\right)$$

where 

$a=\frac{\sigma_{\alpha }^{2}+\sigma_{\beta }^{2}}{2\sigma_{\alpha }^{2}\sigma_{\beta }^{2}},b=\frac{\sigma_{\alpha }^{2}-\sigma_{\beta }^{2}}{2\sigma_{\alpha }^{2}\sigma_{\beta }^{2}},\;c=\frac{r}{\sigma_{\alpha }\sigma_{\beta }}$, $r=\frac{\sigma_{\alpha \beta }}{\sigma_{\alpha }\sigma_{\beta }}$; $\sigma_{\alpha }$, $\sigma_{\beta }$—standard deviations of the real and imaginary part of the voltage, respectively; *r*—correlation coefficient between the real and imaginary parts; $\sigma_{\alpha \beta }$—covariance; $I_{0}$—modified zero order Bessel function.

**Figure 1:**
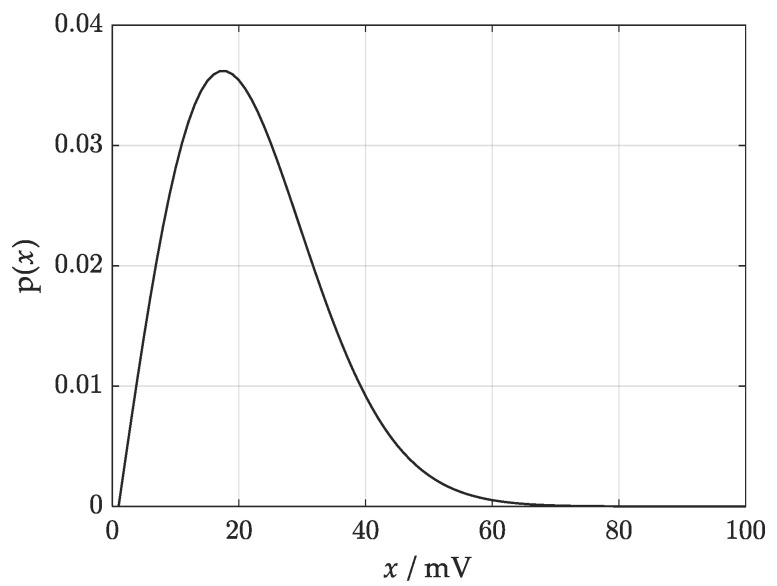
Generalized Rayleigh distribution characterizing measurand.

Equation (4), derived in [22], is a generalized Rayleigh distribution, adequate for the measured voltage magnitude, for which the real part and imaginary part are characterized by normal probability distributions with zero mean values and various standard deviations. Figure 1 shows the graph of the probability density function (4) for the given standard deviations: $\sigma_{\alpha }$ = 14.8 mV, $\sigma_{\beta }$ = 18.6 mV, and correlation coefficient *r* = 0.

The measured voltage magnitude *x* is a de-stimulant of quality and too high values speak against the product. The binary classification of product items, distinguishing between correctly made (conforming with specification) items and items that exceed the specified voltage magnitude level (non-conforming), is performed by direct comparison with the upper acceptance limit $D_{u}$ using an analog comparator, for which we know the standard uncertainty of the threshold. Distribution of the comparator threshold depends on stochastic and deterministic effects. It represents stochastic temporal noise comprising thermal and flicker noise, and the deterministic offset due to fabrication imperfection and transistor mismatch. It was found empirically that the distribution of the comparator threshold is Gaussian [36].

The observation of the measurand *x* is disturbed by the random component *W*, representing the uncertainty of the comparator threshold

(5) Y=X−W

The sensor process is described by the conditional probability distribution *p*(*y*|*x*), which is the *Y* distribution for *X* taking a fixed value of *x*. When we substitute *X = x* into (5), the random variable *Y* differs from *W* by only the fixed component of *x*. Hence, the *Y* distribution for a fixed *X* is the *W* distribution which we will denote $p_{W}\left(.\right)$. Thus, we have

(6) $$p\left(y|x\right)=p_{W}\left(x-y\right)$$

**Figure 2:**
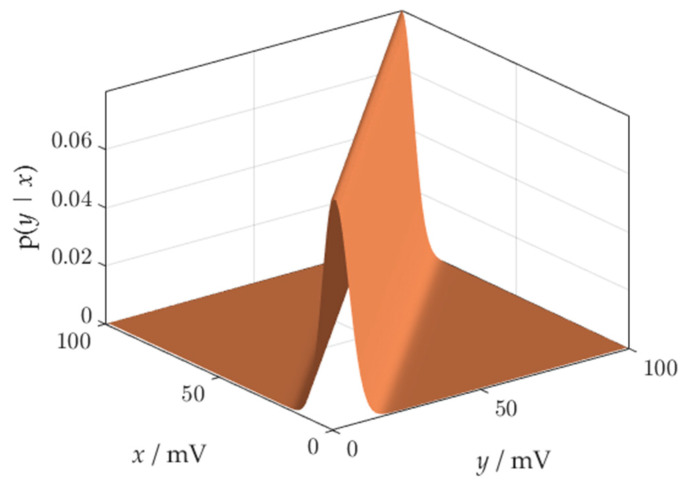
A probability density function governing the sensor process.

If we use the normal distribution to characterize the uncertainty of the comparator threshold, then the conditional probability distribution *p*(*y*|*x*), describing the sensor process, has the shape shown in Figure 2 (standard deviation of the distribution is equal to 5 mV).

**Figure 3:**
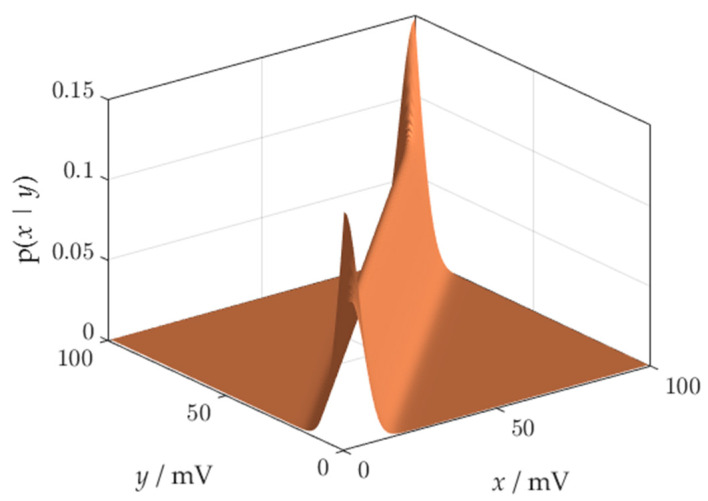
A result of probabilistic inversion.

The reconstruction process is the numerical transformation that maps the observation into the measurement value. It is the stage of information processing which, depending on the technology, can be embedded in the measuring system, or implemented off-line [9]. The reconstruction is based on the probabilistic inversion of distribution (6). The result of the inversion is presented in Figure 3.

The pdf obtained after probabilistic inversion (Figure 3) is distorted at the ends of the measuring range due to the boundary effect, which will be described in Section 5.1. Distortions are a significant source of errors in risk assessment.

We will obtain a probabilistic model of the overall measurement process by combining the sensor process with the reconstruction process using (3). Result has the form of a conditional probability distribution, combining every possible value of the measurand with the possible measurement results that can be attributed to the measurand. Concatenation is another source of distortions at the ends of the measurement range.

The synthesis of the joint probability distribution of the measured value $x_{m}$ and the measurand *x* requires the multiplication of the distribution characterizing the measurement process, and the distribution characterizing the production process. The probability distribution *p*(*x*) does not depend upon the probability distribution characterizing the measurement equipment, and vice versa, the model of measurement process $p\left(x_{m}|x\right)$ does not depend on the probability distribution characterizing the measurand. Hence, the distributions listed are statistically independent, their product forms the joint distribution of the measurement results and the measurand

(7) $$p\left(x_{m},x\right)=p\left(x_{m}|x\right)p\left(x\right)$$

**Figure 4:**
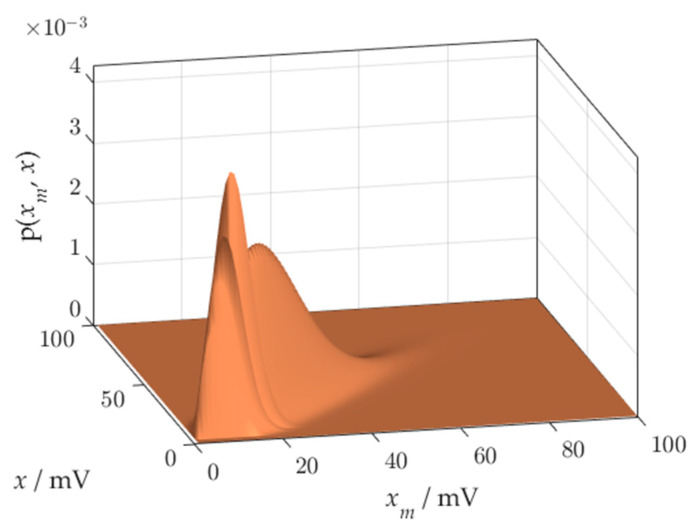
Joint pdf of measurement value *x_m_* and measurand *x*.

The joint pdf of the measurement results $x_{m}$ and the measurand *x* is presented in Figure 4. Distortion of the distribution body near the beginning of the measuring range can be observed.

**Figure 5:**
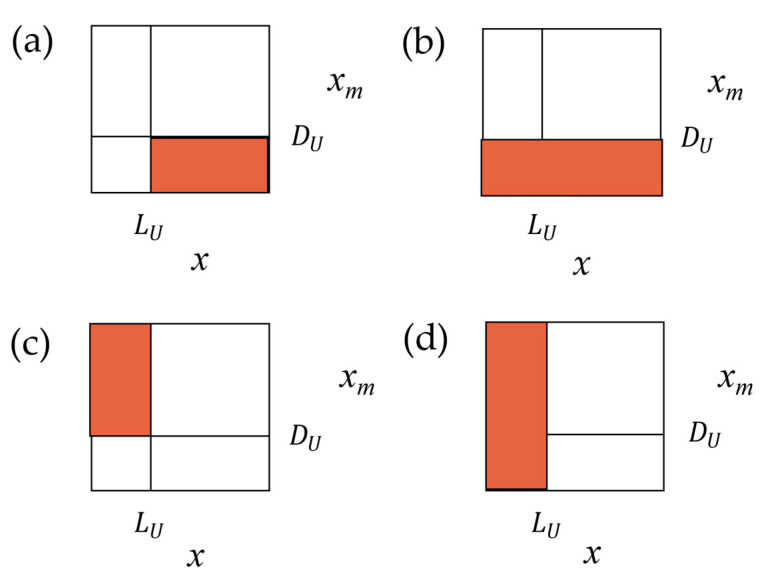
Specified upper tolerance limit *L_U_* and upper acceptance limit *D_U_* defining integration areas for calculation of the probability that: (**a**) an item that passes the test, in fact, does not conform to the specification; (**b**) an item will be accepted; (**c**) an item is truly functional, but it fails the test; (**d**) an item is truly functional.

The risk metrics, presented in Section 2, are useful for testing the probabilistic model of a measurement process. The values of consumer’s and producer’s risks can be calculated based on distribution (7) after determining the appropriate integration areas. This task is facilitated by Figure 5, which shows the square areas, determined by the range of measurand changes, and the domain of the measurement results, both from 0 mV to 100 mV. The vertical line corresponds to the specified upper tolerance limit $L_{u}$. The horizontal line corresponds to the upper acceptance limit $D_{u}$. The values $L_{u}$ and $D_{u}$ do not have to be equal; often a margin is used between them to manage the risk. The probability for a given rectangular region is found by integrating the joint pdf (7) over the region. Working formulas for determining individual risk types can be derived by using Figure 5. Figure 5a shows the shaded region used to calculate the probability of false acceptance of the non-conforming product. The region in Figure 5b allows one to calculate the probability that the product will be accepted. The probability of rejecting a product that conforms to the specification is represented by the shaded region in Figure 5c. The shaded region in Figure 5d allows one to calculate the probability that the product is truly functional.

To calculate the risk metrics: false acceptance and false rejection, based on the joint probabilities, we use the following equations:

(8) $$P\left(A,B\right)=\int_{x_{m}\leq D_{U}}\int_{x>L_{U}}p\left(x_{m},x\right)dxdx_{m}$$

(9) $$P\left(R,G\right)=\int_{x_{m}>D_{U}}\int_{x\leq L_{U}}p\left(x_{m},x\right)dxdx_{m}$$

To determine the risk metrics based on the conditional probabilities—*P*(*A|B*), *P*(*B|A*)*, P*(*R|G*)*,* and *P*(*G|R*)—we divide the surface areas of the respective regions of Figure 5 as follows:

(10) $$P\left(A|B\right)=\frac{\int_{x_{m\leq D_{U}}}\int_{x>L_{U}}p\left(x_{m},x\right)dxdx_{m}}{\int_{x_{m}}\int_{x>L_{U}}p\left(x_{m},x\right)dxdx_{m}}$$

(11) $$P\left(B|A\right)=\frac{\int_{x_{m}\leq D_{U}}\int_{x>L_{U}}p\left(x_{m},x\right)dxdx_{m}}{\int_{x_{m}\leq D_{U}}\int_{x}p\left(x_{m},x\right)dxdx_{m}}$$

(12) $$P\left(R|G\right)=\frac{\int_{x_{m}>D_{U}}\int_{x\leq L_{U}}p\left(x_{m},x\right)dxdx_{m}}{\int_{x_{m}}\int_{x\leq L_{U}}p\left(x_{m},x\right)dxdx_{m}}$$

(13) $$P\left(G|R\right)=\frac{\int_{x_{m}>D_{U}}\int_{x\leq L_{U}}p\left(x_{m},x\right)dxdx_{m}}{\int_{x_{m}>D_{U}}\int_{x}p\left(x_{m},x\right)dxdx_{m}}$$

For example, to calculate the *P*(*R|G*) risk, we used the probability that the conforming product will fail the test, calculated using the region from Figure 5c, and the probability that the product conforms to the specification, determined using the region from Figure 5d.

The risk metrics can be used, among others, in product quality control, test design, and fault diagnosis. An example of practical application is evaluation of test quality and comparison of test architectures for fully differential electronic circuits, by using two risk metrics: *P*(*B*|*A*) as defect level and *P*(*R*|*G*) as yield loss [22].

## 5. How to Apply the Probabilistic Model of Measurement Process Successfully

This section raises a variety of practical points on modeling considerations, in particular, the necessity of using the likelihood function and cautions regarding the choice of domain during calculation of the joint pdf $p\left(x_{m},x\right)$.

### 5.1. Application of the Likelihood Function

The inversion of the conditional probability distribution *p*(*y*|*x*) is realized by using Bayes’ Formula (2) but there is no mechanism by which the x- and y-axes are transposed to obtain the correctly oriented posterior distribution *p*(*x*|*y*). This problem was solved by R.A. Fisher, who proposed the concept of likelihood. Following this concept, we are using the likelihood function instead of *p*(*y*|*x*). Given the data *y*, *p*(*y*|*x*) may be regarded as a function not of *y* but of *x*. It is called the likelihood function of *x* for a given *y* and can be written as

(14) p(y|x)=L(x;y)

As a result of using the likelihood function, the posterior conditional pdf is correctly oriented in space.

The likelihood function (14) looks like the joint probability, but a semicolon is used in its notation instead of a comma, which means that *y* is not a random variable but a parameter. From a geometric standpoint, the family of probability distributions *p*(*y*|*x*) can be viewed as a family of curves parallel to the y-axis, while the family of likelihood functions L(*x*; *y*) are the curves parallel to the x-axis. Construction of an appropriate function L(*x*; *y*) requires engineering knowledge which is specific to the measuring process being modeled. Box and Tiao [37] described a method based on a data-translated likelihood. Mathematically, the data-translated likelihood must be expressible for a given *x* and parameter *y* in the data translated format

(15) L(x;y)=φ[f(x)−t(y)]

Here, the likelihood is the function L = *φ*(*z*), where *z* = *f*(*x*) − *t*(*y*). In the case of Gaussian function, for the mean, f(x)=x, and *t*(***y***) $=\bar{y}$. For the standard deviation f(σ)=logσ, and *t*(***y***) = log s. In our case, parameter *y* is scalar, hence in Matlab format, concrete form of the likelihood function for the mean is

L(*x*; *y*) = ((1./sqrt(2. ∗ pi. ∗ *u*.^2)). ∗ exp(−((*x* − *y*).^2)./2./*u*.^2))

where *u* is the measurement uncertainty.

**Figure 6:**
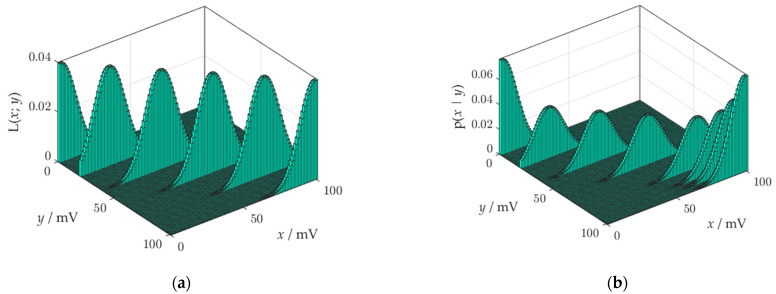
Likelihood functions: (**a**) before standardization; (**b**) after standardization.

In the data-translated likelihood function, *y* only influences *x* by a translation on the scale of the function *φ*, i.e., from *φ*(*z*) to *φ*(*z* + *a*). To put it simply, the data only serve to change the location of the likelihood function (Figure 6a). The likelihood functions have the same form for each observation *y*, but those near the ends of the observation domain are cut off.

Using the likelihood function and the uniform prior, we can write Bayes’ Formula (2) as

(16) $$p\left(x|y\right)=\frac{L\left(x;y\right)}{\int_{L_{x}}L\left(x;y\right)dx}$$

if the integral $\int_{L_{x}}L\left(x;y\right)dx$, taken over the admissible range of *x*, is finite.

The role of the denominator in Bayes’ formula (16) is the standardization of the likelihood function. The standardization means the likelihood is scaled so that the area of each section is equal to one. Apart from standardization, nothing is performed by Equation (16). Hence, the posterior distribution is equal to the standardized likelihood function. A side effect of the standardization are distortions of the posterior at the ends of the observation domain, which can be the source of errors during further calculations. The mechanism of formation of distortions is shown in Figure 6b. Likelihood functions that have a cropped surface area, during standardization must be lengthened up for the surface area to reach a value of 1. The probabilistic inversion distorts the posterior at the edges of the observation domain more, the greater the measurement uncertainty. This is a significant disadvantage of the model of reconstruction sub-process.

**Figure 7:**
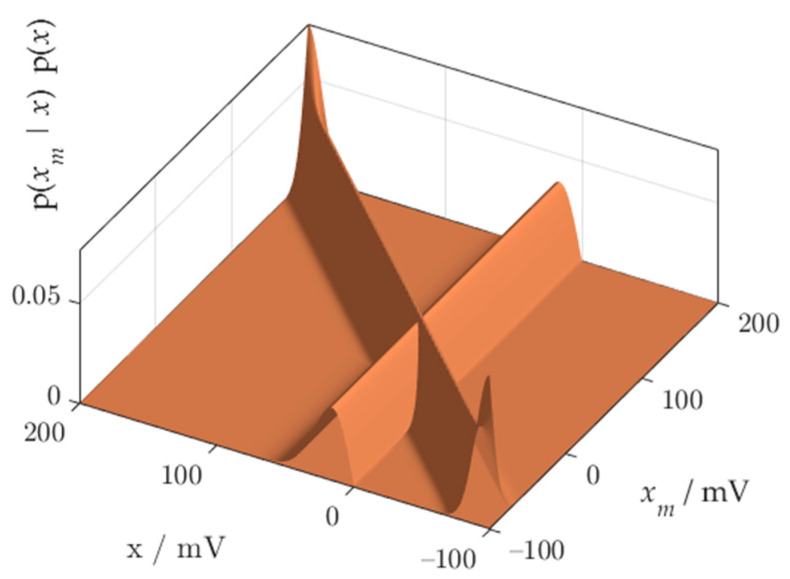
Calculation of the joint pdf using the extended domains of measurand and measurement values.

The method of correct calculation of the joint pdf is based on the extension of the x and $x_{m}$ domains. We observe the joint distribution $p\left(x_{m},x\right)$ in the nominal domain, looking for distortions or truncation. The distribution should be observed at the ends of the measuring range. The elimination of distortions consists in extending the domain until the results of the risk calculations stop changing. To avoid the influence of distortion on the result of the multiplication of probabilities *p*(*x*) and $p\left(x_{m}|x\right)$, the pdfs should cross each other in the area free from distortion. In the case of Rayleigh distribution, this means that during calculations of the joint pdf, the domains of measurand x and measurement value $x_{m}$ should be extended below the 0 point of the measuring range. Figure 7 shows an example of the extended x and $x_{m}$ domains, where the distortions do not spoil the result of multiplication of two distributions.

### 5.2. The Choice of the Domain of Measurement Values during Calculations of the Joint pdf $p\left(x_{m},x\right)$.

**Figure 8:**
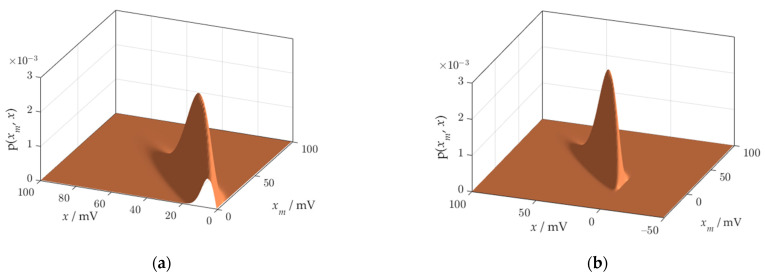
Joint probability density function $p\left(x_{m},x\right)$ obtained using the probabilistic model: (**a**) clipped at the zero point of the domain of $x_{m}$; (**b**) reconstructed thanks to the widening the domain of $x_{m}$.

Another important point in risk calculation is the choice of the domain for $p\left(x_{m},x\right)$. The product of the Rayleigh and Gauss densities goes below the zero point of the domain of measurement values, in the direction of negative values of $x_{m}$. The effect of exceeding the zero point is strengthened as the measurement uncertainty increases. A truncation appears (Figure 8a), which is the source of errors during integration of the volume under the surface of the solid figure of the joint probability density.

The accuracy of the risk metrics obtained with the probabilistic model can be greatly increased by expanding the domain of measurement results $x_{m}$ below the measurement range. As a result, the joint probability density will be formed in full (Figure 8b).

### 5.3. Testing of the Model

The results of testing the model by using the risk metrics specified in Table 1, Table 2 and Table 3, are summarized in Table 4. Tests were performed for standard measurement uncertainties equal to u = (2, 5, 10) mV and 6 metrics of risk of erroneous decisions, resulting from these measurement uncertainties, and calculated using (8–13).

The following column labels are used in Table 4: Model 1—risk metrics based on the model presented in Section 3, Model 2—risk metrics based on the model, in which the domains of measurand and measured values have been extended, MC—risk metrics obtained by the Monte Carlo method.

Table 4 allows us to evaluate the conformity of the risk metrics obtained using the analytical model with the reference risk metrics obtained by the MC method. The results of the MC analysis confirmed the correctness of the Model 2-driven calculations.

For the moderate measurement uncertainty (*u* = 5 mV), the biggest error of determination of the risk *P*(*R*|*G*) by using Model 1, relative to the reference MC method, reaches 3%. As the measurement uncertainty increases, Model 1 generates larger errors of risk assessment. For example, the assessment error of the risk *P*(*B*|*A*), for measurement uncertainty *u* = 10 mV, reaches even 9% of the reference value. Risk assessment errors are the result of the distortions generated during calculation steps and are the result of truncation of the joint probability distribution. The location of the Rayleigh distribution at the beginning of the measuring range, in the area of distortions, favors errors.

A significant improvement in the conformity of the analytical results with the MC method can be observed for Model 2. Conformity was obtained because the joint probability distribution is free from distortion and truncation.

In the Monte Carlo method, samples from the generalized Rayleigh distribution, adequate for the measured voltage magnitude, are obtained by geometric summation samples of the real part and imaginary part, generated by the normal probability distributions with zero mean values and various standard deviations. The uncertainty *u* is entered in the simulation as the standard deviation of the normal distribution, characterizing the sensor sub-process. The generated pseudo-random number, with standardized normal distribution, is multiplied by *u*. The resulting pseudo-random number "*offset*" is added to the "*threshold*" of the comparator. An excerpt from the Matlab program demonstrates the simplicity of this approach:

N = 1000000000;

R = sqrt(normrnd(*mualfa*,*sigmaalfa*,N,1).^2 + normrnd(*mubeta*,*sigmabeta*,N,1).^2);

offset = *u*.*randn(N,1);

for k = 1:N;

if (le(R(k), *threshold* + *offset*(k)))&(gt(R(k), *threshold*))

counter = counter + 1;

end

The reliability of the reference model was assessed using confidence intervals. Table 5 presents examples of confidence intervals expressed in ppm for the two most popular risk measures in the context of production processes—defect level and yield loss. Risk measures are mean values of 30 random populations of 10^9^ instances. The analysis of Table 5 leads to the conclusion that only 2 or 3 digits of the risk assessment results are stable. However, the practical use of the results requires a larger number of digits, which implies a larger number of trials than the applied 10^9^, and hence the usefulness of the MC method is limited. The following practical example illustrates this problem and shows the advantage of the analytical model over the MC method.

### 5.4. Validation of the Correctness and Consistency of the Testing Results

To assess the practical usefulness of the results collected in Table 4, let us consider an example of a production process, characterized by low yield: about 94%. For production characterized by the probability distribution in Figure 1, we get this production yield for a measured voltage upper tolerance limit $L_{u}$ = 40 mV. From (4), we calculate that the probability of producing a conforming product is 93.91604%. In a batch of one million copies, 939,160 products meet the specification (this is, the production yield), and 60,840 do not conform. Let us assume that to detect and eliminate the non-conforming products, we test the voltage amplitude with a comparator, whose threshold uncertainty equals *u* = 5 mV and the value of the voltage upper acceptance limit $D_{u}$ = 40 mV. In the middle column of Table 4, for Model 2 we read the value of *P*(*R*,*G*) = 2.34580%. This means that because of the uncertainty of measurements, 23,458 eligible products will be incorrectly classified as non-conforming. Hence, 93,9160 – 23,458 = 915,702 products will be qualified as conforming. The value of *P*(*A*,*B*) = 1.17412% indicates that due to the uncertainty of measurements, an additional 11,741 non-conforming products will be incorrectly qualified as conforming. In total, 915,702 + 11,741= 927,443 products will enter the market, of which 11,741/927,443 ≈ 1.2659% will be unsuitable for use. This value corresponds to a consumer risk level, determined in accordance with the definition of *P*(*B*|*A*) = 1.2660%. The ratio of the number of incorrectly rejected products to the number of products constituting the production yield will be 23,458/939,160 ≈ 2.4977%, which corresponds to the producer’s risk level, determined by *P*(*R*|*G*) = 2.49771%. Analysis of this example production process confirms the mutual agreement of the results of the risk calculations presented in the middle column of Table 4. Similar calculations carried out for the other two uncertainties: *u* = 2 mV, and *u* = 10 mV, confirmed the correctness and consistency of the results throughout Table 4, as well as the practical usefulness of the proposed way of modeling.

## 6. Discussion

Measurement results are accompanied by their uncertainty, which carries the risk of making erroneous decisions in accepting or rejecting a product. Risk analysis resulting from measurement uncertainty is used, among others, in the design of tests to check the compliance of products with the specification. It enables the determination of the required accuracy for measuring instruments, and the selection of the acceptance range for the tested product attribute (risk management). It also allows the impact of measurement uncertainty on direct production costs, and on the costs of the consequences of incorrect decisions of conformity, to be assessed.

The paper presents problems arising during the application of the probabilistic model of measurement process to analyze the risk of erroneous decisions in conformity assessment and proposes ways to solve them. It was found that in Bayes’ formula, used for probability inversion, there is no mechanism for the correct orientation in space of the posterior distribution, and hence the need to use the likelihood function arises.

The use of multiple risk metrics allowed thoroughly examine the model, because each fragment of the two-dimensional probability distribution was investigated during calculations. The procedure for calculating risk using the model is complex. In the stages of calculations, distortions arise that must be monitored and eliminated by selecting the domain. The first source of distortion is inversion of the conditional distribution. The second, independent source of distortion is the concatenation of two sub-processes. The third source of error is truncation of the joint distribution. The effect of truncation of the solid figure of a joint probability distribution $p\left(x_{m},x\right)$ is the result of exceeding the zero point of the domain of measured values. In-depth study of probabilistic model requires experimenting with probability distributions of various shapes. The truncated join distribution was detected due to the shape of the generalized Rayleigh distribution that was used in the case study. It is possible to reconstruct the distribution body by performing calculations, in a domain of measured values $x_{m}$, wider than the measuring range. This remark is especially important when the measurement process is affected by high uncertainty.

## 7. Conclusions

The main contribution of the work includes:

- A proposal to test a probabilistic model of the measurement process by six metrics of risk in parallel.
- Detection of the distortion and truncation effect of the joint probability distribution *p*(*x_m_*, *x*), which is the basis for calculation risk metrics.
- Giving a recommendation on how to avoid distortion and clipping of the distributions.
- Obtaining compliance of the analytical results with the results of Monte Carlo simulation.

## Figures and Tables

**Table 1:** Joint probabilities characterizing the binary decision process.

Test Result	Status of an Item under Test	
	Good (*G*)	Bad (*B*)
*A*	Valid acceptance $P\left(A,G\right)=P\left(x\in L,x_{m}\in D\right)$	False acceptance $P\left(A,B\right)=P\left(x\notin L,x_{m}\in D\right)$
*R*	False rejection $P\left(R,G\right)=P\left(x\in L,x_{m}\notin D\right)$	Valid rejection $P\left(R,B\right)=P\left(x\notin L,x_{m}\notin D\right)$

**Table 2:** Probabilities conditioned on the status of an item under test.

Test Result	Status of an Item under Test	
	Good (*G*)	Bad (*B*)
*A*	Good item accepted $P\left(A|G\right)=\frac{P\left(x\in L,x_{m}\in D\right)}{P\left(x\in L\right)}$	Bad item accepted $P\left(A|B\right)=\frac{P\left(x\notin L,x_{m}\in D\right)}{P\left(x\notin L\right)}$
*R*	Good item rejected $P\left(R|G\right)=\frac{P\left(x\in L,x_{m}\notin D\right)}{P\left(x\in L\right)}$	Bad item rejected $P\left(R|B\right)=\frac{P\left(x\notin L,x_{m}\notin D\right)}{P\left(x\notin L\right)}$

**Table 3:** Probabilities conditioned on the test results.

Test Result	Status of an Item under Test	
	Good (*G*)	Bad (*B*)
*A*	Accepted item good $P\left(G|A\right)=\frac{P\left(x\in L,x_{m}\in D\right)}{P\left(x_{m}\in D\right)}$	Accepted item bad $P\left(B|A\right)=\frac{P\left(x\notin L,x_{m}\in D\right)}{P\left(x_{m}\in D\right)}$
*R*	Rejected item good $P\left(G|R\right)=\frac{P\left(x\in L,x_{m}\notin D\right)}{P\left(x_{m}\notin D\right)}$	Rejected item bad $P\left(B|R\right)=\frac{P\left(x\notin L,x_{m}\notin D\right)}{P\left(x_{m}\notin D\right)}$

**Table 4:** Results of the risk analysis for Model 1, Model 2, and results of the MC simulation for the measurand with the Rayleigh distribution and the Gauss distribution characterizing the measuring system.

Risk Metric	Measurement Uncertainty (mV)								
	*u* = 2			*u* = 5			*u* = 10		
	Risk of Incorrect Decision (%)								
	Model 1	Model 2	MC	Model 1	Model 2	MC	Model 1	Model 2	MC
*P*(*A*, *B*)	0.5732	0.57109	0.570	1.1790	1.17412	1.174	1.7767	1.76977	1.768
*P*(*R*, *G*)	0.7606	0.75781	0.757	2.3547	2.34580	2.344	6.3781	6.35267	6.356
*P*(*A*|*B*)	9.3824	9.38061	9.380	19.298	19.2979	19.30	29.084	29.0757	29.07
*P*(*B*|*A*)	0.6139	0.61034	0.609	1.3010	1.26600	1.266	2.1615	1.98115	1.980
*P*(*R*|*G*)	0.8131	0.80506	0.806	2.5651	2.49771	2.496	7.3484	6.76429	6.768
*P*(*G*|*R*)	12.080	12.0771	12.07	32.323	32.3274	32.32	59.552	59.5563	59.56

**Table 5:** The confidence intervals of the risk metrics reference values.

Risk Metric	Measurement Uncertainty (mV)		
	*u* = 2	*u* = 5	*u* = 10
	Confidence Interval (ppm) for Confidence Level of 95%		
*P*(*B*|*A*)	6092 ± 5	12,654 ± 7	19,802 ± 9
*P*(*R*|*G*)	8062 ± 6	24,969 ± 10	67,668 ± 16

## Data Availability

Data sharing is not applicable to this article.
